# Infection-targeted innovations in anastomotic leak management: endoscopic advances, antimicrobial biomaterials, and precision strategies

**DOI:** 10.1080/07853890.2026.2619218

**Published:** 2026-01-23

**Authors:** Yansong Xu

**Affiliations:** Department of Emergency, The First Affiliated Hospital of Guangxi Medical University, Nanning, Guangxi Zhuang Autonomous Region, P.R. China

**Keywords:** Anastomotic leakage, antibacterial hydrogel, endoscopic vacuum therapy, hospital-acquired infections, precision medicine

## Abstract

Anastomotic leakage (AL) is a devastating complication of gastrointestinal surgery and a critical source of hospital-acquired infections. This review synthesizes recent advances in AL management, with a focus on infection-targeted strategies. A narrative review of the literature was conducted, encompassing established clinical therapies and emerging preclinical innovations. For mature endoscopic modalities, such as Endoscopic Vacuum Therapy (EVT), clinical cohort data and meta-analyses support high fistula closure rates (74–94.4%) and a significant reduction in sepsis risk. In parallel, novel preclinical strategies—including antibiotic-eluting hydrogels, reversible endoscopic bypass procedures, and artificial intelligence-driven prediction models—demonstrate transformative potential in early-stage studies but require robust clinical validation. The future management of AL is evolving towards an integrated model, combining evidence-based endoscopic interventions with intelligent biomaterial-based approaches. Translating preclinical innovations into practice will necessitate overcoming challenges related to cost, standardization, and long-term safety, ultimately enabling a paradigm shift from reactive repair to proactive, precision prevention.

## 1. Introduction

AL is a critical portal for hospital-acquired infections, with 24–38% of patients developing incisional infections and 16–24% progressing to sepsis [1,2]. Among the most severe complications following gastrointestinal surgery, AL refers to the leakage of non-sterile gastrointestinal contents into the peritoneal cavity, leading to infection, sepsis, or even multiple organ failure. Conventional management strategies include conservative approaches (e.g. fasting, drainage, nutritional support), endoscopic interventions, and surgical repair. The development of novel biomaterials and emerging technologies has demonstrated efficacy in reducing AL rates [3,4]. Intelligent therapeutic approaches represent an evolving frontier, utilizing robotic platforms and decision-support systems to establish closed-loop ‘monitoring-alert-intervention’ frameworks that mitigate reoperation risks.

This review aims to synthesize the evolving landscape of AL management by critically analyzing two interconnected domains: (1) established and emerging clinical therapies that are reshaping minimally invasive intervention, and (2) preclinical and translational innovations in antimicrobial biomaterials and precision strategies that hold promise for transforming prevention, diagnosis, and treatment. By juxtaposing current standards with frontier technologies (Table 1), this review seeks to provide a comprehensive roadmap that informs clinical practice while highlighting the translational pathways required to integrate future breakthroughs into patient care.

**Table 1.** Literature search strategy.

Concept A (Disease)	Terms related to anastomotic leak (‘Anastomotic Leak’ [Mesh], ‘anastomotic dehiscence’, ‘suture line leak’).
Concept B (Infection)	Terms related to infection and antimicrobial approaches (‘Surgical Wound Infection’ [Mesh], ‘Sepsis’, ‘anti-bacterial agents’).
Concept C (Innovations)	Terms covering the review’s focus areas: endoscopic therapy (‘Endoscopic Vacuum Therapy’, ‘stents’), biomaterials (e.g. ‘coated sutures’, ‘antimicrobial hydrogel’), and precision strategies (e.g. ‘Indocyanine Green’, ‘perfusion imaging’).
Final search equation= ‘Concept A’ and ‘Concept B’ and ‘Concept C’	

## 2. The harmfulness of gastrointestinal AL

AL exhibits marked incidence variability across gastrointestinal surgeries, influenced by operative techniques, patient comorbidities, and perioperative management [5–7]. This complication carries grave consequences: mortality escalates to 35% in uncomplicated cases and exceeds 80% when complicated by septic shock [5]. The economic burden is substantial, with mean treatment costs of €57,000 in European cohorts – severe AL incurs approximately triple the costs of mild cases (€126,000 vs. €36,000; *p*<0.001) [2,8]. Furthermore, 24–32% of patients require reoperations [1,8,9], while 15–40% develop chronic anastomotic strictures or permanent bowel discontinuity, collectively contributing to lifelong functional impairment and diminished quality of life [10].

## 3. Traditional treatment strategies for gastrointestinal AL

Over the past three decades, therapeutic strategies for anastomotic leakage have undergone significant evolution in response to this formidable challenge. Initial management centered on direct surgical repair, resection of the affected bowel segment, or diversion stoma creation. However, these invasive approaches carry substantial morbidity, including high rates of incisional infection (26–38%) and intra-abdominal infection (74%) [1,8,9]. Consequently, for selected cases involving well-contained fistulas, the paradigm has shifted towards supportive management. This strategy employs bowel rest, total parenteral nutrition support, and intensive antibiotic therapy to control infection, achieving success rates exceeding 80% [9,11,12]. While supportive therapy avoids the trauma of reoperation, it necessitates a protracted healing period, often requiring months of fasting and nutritional support.

Supportive management is primarily indicated for patients with anastomotic leakage presenting with localized clinical manifestations and no systemic signs of infection. A global multicenter study involving 71 centers suggested that a more conservative initial approach for select presentations of anastomotic leakage may potentially reduce complication rates [8]. Evidence indicates that for post-esophagectomy anastomotic leakage with localized features (e.g. absence of intrathoracic fluid collections and adequate graft perfusion), endoscopic interventions may yield superior outcomes compared to supportive management alone [8]. In cases complicated by a localized abscess cavity, drainage procedures (e.g. percutaneous or endoscopic drainage) combined with fistula closure techniques are prioritized over extensive surgical intervention [8,9]. Following rectal cancer surgery, anastomotic leakage can often be effectively managed by fecal diversion (e.g. stoma creation) combined with drainage to control infection [13]. Supportive therapy typically encompasses antibiotic administration, nutritional support and close monitoring. For patients with upper gastrointestinal anastomotic leaks, jejunostomy is a common route for providing enteral nutritional support [11]. The severity of anastomotic leakage, such as assessed by the SEAL score for esophageal leaks [2],may influence the selection of nutritional strategies. However, standardized protocols for nutritional assessment and supplementation remain lacking, particularly in patients at high risk for anastomotic leakage [14].

The SEAL scoring system, developed from a multicenter retrospective study (71 centers, 2011–2019), integrates critical prognostic factors such as systemic infection, intrathoracic abscess, and anastomotic necrosis. This score objectively quantifies leak severity and predicts 90-day mortality, thereby informing treatment decisions [2]. Surgical intervention is indicated in the following scenarios: 1. Failure of conservative management: Persistent fistula tracts failing to heal after ≥2 weeks of supportive therapy may require endoscopic vacuum therapy (EVT) [11]; 2. Severe clinical manifestations: These include extensive leakage, systemic infection, or intrathoracic abscess, typically necessitating drainage combined with defect closure or drainage alone [8]; 3. High-output conduit leaks (HOCL): Defined as leaks exceeding 1 L/24 h, HOCL are often refractory to conservative management [15]. Reported surgical approaches are diverse, encompassing procedures such as abdominal lavage and drainage, conversion to Roux-en-Y gastric bypass, stoma creation, and fistula resection [16]. For postoperative anastomotic leakage following rectal cancer surgery, a large-scale global cohort study (216 centers) classified surgical strategies into categories including salvage surgery and fecal diversion with or without repair. Surgical decision-making should be guided by leak severity [9] While protective stoma creation was associated with a reduction in anastomotic leakage rates (from 7.5% to 5.5%) in one study, this difference was not statistically significant (*p* = 0.78) [6]. Salvage surgery may be necessary for high-risk patients (e.g. those with significant intraoperative blood loss or undergoing lymph node dissection), though it requires careful consideration of long-term survival implications [1]. Regarding delayed intestinal reconstruction, consensus is lacking on whether hand-sewn anastomosis (single-layer/double-layer) or stapled anastomosis (circular/linear stapler) is associated with a lower risk of AL [17].

## 4. The rise of minimally invasive intervention technology: current status and evidence based approach

In recent years, minimally invasive interventions have rapidly emerged as a core strategy in the management of AL, driven by their advantages of reduced trauma and accelerated recovery. To provide clear clinical guidance, we categorize these modalities based on their translational maturity and level of evidence. The following sections will first discuss established clinical therapies, followed by emerging experimental technologies.

### 4.1. Established endoscopic clinical modalities

The rapid advancement of endoscopic techniques has brought about transformative changes. Endoscopic modalities such as clip closure (e.g. Over-The-Scope Clip, OTSC) [11], self-expandable metal stents (SEMS) [8], and EVT [12,18–20] have become mainstream approaches. These techniques aim to achieve the ideal therapeutic goals: precise fistula closure, early resumption of oral feeding, and maximal preservation of intestinal function. EVT reduces sepsis risk by 68% in clinical cohorts (OR = 0.32) [12], contrasting with 16.7–24.1% sepsis rates in open surgery [1,8]. The new antibiotic hydrogel further controls the sepsis rate to ≤3.8%, and its local antibacterial effect (such as the anti MRSA efficiency of 99.8%) has critical value in preventing infectious complications [3]. This shift has significantly improved both treatment experience and clinical outcomes for patients (Table 2).

**Table 2.** Comparative outcomes of traditional open surgery vs. minimally invasive interventions for anastomotic leak management.

Parameter	Traditional open surgery	Minimally invasive interventions	Sources	Statistical significance
Reoperation rate	Control: 8.2%	ICG-FI Group: 4.7%	Watanabe [21]	*p* < 0.05
	Conventional surgery: OR = 3.2 (vs. EVT)	EVT Group: Reference	de Lacy [12]	OR = 3.2, 95% CI 2.1–4.9, *p* < 0.001
Treatment cost	Standard cost	EVT reduces costs by 32%	Wannhoff [20]	Significant cost-effectiveness
Complication Risks				
Intra-abdominal infection	74%	Markedly reduced *via* biological barriers	Wang [3]	Mechanistic advantage
Sepsis incidence	Conventional surgery	EVT Group: Reference	de Lacy [12]	Risk of sepsis in EVT group ↓68%, *p* < 0.05
	Conventional surgery (26.9%)	TAAG (0)	Wang [3]	Rat model, *p* < 0.001
Tissue tearing	Rare	↑ Risk with OTSC in fragile tissues	Lee [10]	Technical limitation
Device-related complications	None	OTSC dislodgement: 12.3% (1-year)	Ubels [2]	Requires long-term monitoring
Technical success				
Fistula closure rate	Not directly reported	EVT: 74-94.4% (6 months)	Rodrigues-Pinto [22]	High-quality evidence
	Stoma reversal: 62.1%	EVT + early surgery: 81.2%	de Lacy [12]	*p* = 0.03
Stricture rate	Baseline incidence	Immunomodulatory hydrogels: 0%	Ju [23]	Breakthrough advantage

#### 4.1.1. Endoscopic vacuum therapy (EVT)

EVT has become a critical therapeutic modality for upper gastrointestinal (UGI) anastomotic leaks. Continuous negative pressure removes infected debris and reduces bacterial load (<10^4^ CFU/mL in drain fluid [18]. Hence, it demonstrates high closure rates and shorter treatment durations. This promotes granulation tissue formation and facilitates fistula closure. A single-center study conducted at a tertiary referral center documented the initial clinical experience with EVT for managing post-UGI-surgery anastomotic leaks, confirming its feasibility [19]. Multiple studies report success rates (defined as fistula closure without surgical intervention + infection resolution within 6 months) ranging from 74% to 94.4% [12,18–20,22]. A meta-analysis indicates that the need for reoperation was 3.2 times higher in the conventional surgery group compared to the EVT group (OR = 3.2, 95% CI 2.1–4.9, *p* < 0.001) [12]. While EVT offers minimal invasiveness and significant cost-effectiveness (reducing costs by 32%) [20]. EVT is primarily indicated for leaks following UGI procedures and pelvic anastomoses [19,20]. For persistent fistulas refractory to conservative management (e.g. unhealed leaks ≥2 weeks post-esophagectomy), and is preferred over open surgery in patients with hemodynamic instability. The therapy necessitates multiple endoscopic interventions for sponge changes and exhibits high technical dependency. It carries a considerable risk of complications, with anal discomfort reported in up to 37% of cases, particularly when applied for low pelvic leaks [12].

#### 4.1.2. Endoscopic clamping technique

This technique achieves reliable fistula closure through mechanical apposition of tissue edges. Innovations include: Over-the-scope clips (OTSC) and through-the-scope clips (TTSC/Repositionable clips): Utilize compressive force and, in some designs (e.g. MANTIS Closure Device), integrated sharp tines for rapid, secure sealing. Modified double-layer technique: Enhances success through layered closure, ideal for larger or complex defects. Endoscopic suturing/Spiral TTSS: Provides solutions for very large (>2 cm) or irregular perforations by approximating tissue.

Supported by clinical studies demonstrating efficacy. The modified double-layer technique is reported as particularly effective for specific defects [24]. OTSC is widely used, though long-term data show a 1-year dislodgement rate of 12.3% in the UGI tract [8]. Novel systems like TTSS show promise in reducing stricture rates but are associated with higher costs [25]. Esophageal leaks/perforations <2 cm: TTSC or OTSC are recommended. Large colorectal ESD defects and complex anastomotic leaks: Modified double-layer clip techniques or OTSC. Perforations >2 cm: Endoscopic suturing or novel spiral TTSS are viable options. It is also part of combination strategies (e.g. with EVT) to improve outcomes like stoma reversal [12,19]. Technical and device-specific challenges exist: Tissue integrity risk: Clips with sharp tines may cause tissue tearing in fragile (e.g. post-radiotherapy) areas [10]. Anatomic constraints: OTSC deployment can be complex in rectal strictures or angulated segments [17], and its large diameter may increase procedural difficulty. Device-related issues: Risk of clip dislodgement (OTSC) and potential for foreign body reactions in infected sites (e.g. TTSS) [25]. Economic factor: Advanced systems like TTSS are associated with higher costs compared to standard clips [25].

## 5. Emerging and experimental endoscopic strategies

### 5.1. Reversible endoscopic gastric bypass (REGB) surgery

The core mechanism of REGB is to create gastrojejunostomy under endoscopic guidance (using lumen aligned metal stents, LAMS), followed by closure of the pylorus through endoscopic suturing to achieve complete duodenal diversion. This process diverts gastric contents rich in stomach acid from the diseased duodenum, reducing the stimulation of the acidic environment on the fistula opening and promoting tissue healing. This technology is a single, two-step operation with reversibility (the stent can be removed later) [25]. REGB represents a novel endoscopic technique designed to manage postoperative gastrointestinal anastomotic leaks, particularly duodenal leaks and refractory postoperative fistulas. By achieving complete diversion of gastric acid, REGB significantly promotes fistula healing while avoiding the high risks associated with traditional surgery. Preliminary animal studies involving 5 subjects demonstrated a 100% technical success rate [25]. Compared to conventional surgical interventions (e.g. salvage surgery or fecal diversion), REGB offers significantly reduced invasiveness and the potential for shorter hospital stays. While encouraging results have been reported in a small case series (*n* = 6), further validation through larger-scale studies and robust clinical trials is essential to establish its efficacy and safety profile. At present, the evidence focuses on duodenal fistula, while other types of intestinal fistula (such as enterocutaneous fistula ECF) may require different treatment strategies [26]. This technology requires a combination of EUS guided stent placement and endoscopic suturing, which has high technical requirements for the operator [25].

### 5.2. Biomaterials in infection control: antimicrobial mechanisms and clinical evidence

Novel biomaterials offer strategies ranging from adjunctive clinical use to breakthrough preclinical concepts. We distinguish between materials approaching clinical use and those in early-stage development.

#### 5.2.1. Clinically applied and near-clinical biomaterials

The mechanism of self-expandable metal stents (SEMS) treating gastrointestinal fistula includes the following four aspects: reducing fistula output, improving nutritional status, regulating gut microbiota, and optimizing mechanical properties [27]. In terms of technical methods, some studies have used radiofrequency ablation combined with covered metal stent implantation to prolong stent patency time [28]. For complex fistulas, a combination of cavity aligned metal stent and percutaneous covered metal stent can be used [29]. The efficacy data shows that the technical success rate is relatively high in case reports and series studies, and significant effects such as reduced fistula output and improved nutritional status can be seen in clinical improvement [27]. SEMS has clinical value in the treatment of intestinal leakage, especially in malignant diseases and complex fistulas, through mechanical closure and improvement of local environment. However, in terms of long-term effects, there is a possibility of fistula recurrence after stent removal, as well as adverse events such as abdominal pain and fistula formation [30].

#### 5.2.2. Bilayered anti-adhesion patch

The core mechanism of this technology lies in its double-layer structure design, which achieves the function of single-sided adhesion and anti-adhesion on the other side through asymmetric design and biomimetic slurry lubrication and mussel underwater adhesion mechanism [31]. In terms of functional synergy, the adhesive layer promotes healing while preventing anastomotic fistula, while the anti-adhesion layer prevents tissue adhesion by inhibiting fibroblast attachment [3]. In clinical application, its main indication is the prevention of anastomotic leakage and adhesion after gastrointestinal and abdominal surgery Its advantage is that it can simultaneously solve the above two problems and is easy to operator [32]. Animal experiments have shown that its effectiveness is comparable to commercial anti adhesive agents, but clinical data still needs more research to validate it. However, this technology also has several limitations, including insufficient mechanical strength of some patches or poor material absorption, which may affect activity or cause foreign object reactions; Manual placement during surgery is highly dependent and may increase operational difficulty; long term biocompatibility and safety need further evaluation; And currently, research is mostly focused on animal models, with limited human trial data, which poses an important obstacle to clinical translation [33,34].

#### 5.2.3. Preclinical and experimental biomaterial innovations

A novel injectable multifunctional hydrogel (PGOT), composed of γ-polyglutamic acid cross-linked with oxidized hyaluronic acid and tobramycin, demonstrates hemostatic, antibacterial, and pro-healing properties [32]. Immunomodulatory hydrogels (e.g. PHB-gel, MΦ-gel) loaded with factors like IL-4/TGF-β1 reprogram macrophages, promoting angiogenesis and achieving an 88.7% closure rate with zero stenosis [23,35]. However, their long-term immunological risks require validation in large-animal models. Crucially, hydrogel barriers protect anastomoses from corrosive digestive fluids, effectively reducing leakage risk and providing a comfortable alternative to painful nasogastric tube insertion. pH/enzyme-responsive smart hydrogels enable visible color changes within 15 min of leakage, achieving 100% early detection rates [36]. Nevertheless, clinically applicable theranostic materials (e.g. drug-loaded responsive gels) need development (Table 3).

**Table 3.** Findings on novel biomaterials and technologies for treating anastomotic leakage (AL).

Literature	Technology/material	Mechanism of action	Key outcomes	Limitations
Wang et al. [3]	Tissue-Adhesive Antibacterial Hydrogel (TAAG)	Catechol-chitosan network enables wet adhesion; silver nanoparticles provide antibacterial properties	- 92.3% closure rate in rat colon AL model − 99.8% anti-infection efficacy (against MRSA biofilm formation) - Adhesion strength ≥45 kPa	Lacks large-animal studies; high cost ($350/dose)
Yang et al. [37]	Anti-Adhesion Sealing Patch (SandPatch)	Bilayer membrane: silk fibroin/polycaprolactone (inner layer promotes healing; outer layer prevents adhesion)	- Burst pressure ↑180% in porcine intestinal anastomosis - Adhesion incidence ↓91% (vs. control) - Healing time: 14 days (control: 21 days)	Only for intraoperative prevention; ineffective for established fistulas
Ju et al. [23]	Pro-Healing Antibacterial Hydrogel (PHB-gel)	β-glucan gel loaded with IL-4/vancomycin; reprograms macrophages	- 95.6% infection control in rabbit - Angiogenesis density ↑3.2-fold - Complete epithelialization in 28 days	Requires endoscopic precision; IL-4 dosage toxicity unclear
Kang et al. [32]	Double-Layer Bioadhesive	Upper layer: UV-crosslinked gelatin (anti-adhesion); lower layer: Schiff-base reaction (adhesion)	- Leakage pressure: 32.5 mmHg - Adhesion force: 18.9 N/cm² - Antibacterial rate: 99.2% (vs. E. coli)	UV curing limits deep-tissue application
Zhu et al. [35]	Macrophage-Reprogramming Hydrogel (MΦ-gel)	Hyaluronic acid gel loaded with TGF-β1/clindamycin; induces M2 polarization	- 88.7% closure rate in rat colon AL - Collagen deposition density ↑2.8-fold - Stricture rate: 0% (control: 21.4%)	Long-term safety of immunomodulation unverified
Anthis et al. [36]	Smart Hydrogel Sensor	pH/enzyme-responsive hydrogel; colorimetric leakage detection (naked-eye readable)	- 100% early AL detection rate - Response time <15 min - Sensitivity: 94.3%	Monitoring only; no therapeutic function

## 6. Preventive measures for AL

Indocyanine green fluorescence imaging (ICG-FI) significantly reduced AL incidence to 4.2% in minimally invasive rectal cancer surgery. Although its efficacy fell short of anticipated superiority, the ICG+ group demonstrated a significantly lower AL rate (4.7%) versus controls (8.2%), with a reoperation rate of 0.5% compared to 2.4% in controls [21,38]. In addition, thermography generates temperature distribution maps by detecting infrared radiation on the surface of tissues. Areas with good blood circulation usually have higher temperatures, and its advantages are that it does not require exogenous contrast agents and is easy to operate. However, its sensitivity to differences in deep tissues and small blood vessels is limited. Laser Doppler Flow (LDF) and Laser Speckle Imaging (LSI) can provide more direct microcirculation blood flow information. LDF quantifies local blood flow by measuring the Doppler frequency shift of scattered laser from moving red blood cells, but it is usually a single point measurement; LSI can generate large-scale, high-resolution two-dimensional blood flow distribution maps by analyzing the spatiotemporal changes of laser speckle, demonstrating the potential for monitoring blood flow dynamics after intestinal anastomosis in experimental studies. These technologies complement ICG-FI and work together to achieve more objective and accurate anastomotic blood flow assessment during surgery, providing a diverse range of technical options for preventing AL.

## 7. Future direction: precision, intelligence, and integrated treatment

The application of precision medicine in AL management manifests primarily in early precise diagnosis and personalized risk assessment. Regarding diagnostic technologies, the integration of novel biomaterials with advanced imaging techniques shows significant promise [36,39]. Multi-omics technologies provide novel insights for precision prevention of AL. Transcriptomic profiling across different healing phases of anastomotic tissue layers has elucidated molecular signatures of colonic anastomotic healing, laying the foundation for targeted interventions [40].

Artificial intelligence (AI) demonstrates substantial value in AL prediction and management. AI systems enable accurate prediction of esophagogastric/jejunal anastomotic leaks, providing surgeons with real-time risk alerts [41]. Smart drug delivery systems, particularly microneedle-based technologies, are gaining attention due to their minimally invasive nature—ideal for postoperative monitoring and therapy. These systems facilitate real-time wound status monitoring and controlled therapeutic agent release upon demand [42]. Prolonged antibiotic hydrogel use may select for resistant strains. Future biomaterials should incorporate resistance-breaking agents (e.g. quorum-sensing inhibitors [3,32].

Despite significant advances, AL management faces persistent challenges. Technologically, standardized assessment protocols are needed—such as unified indocyanine green fluorescence evaluation—along with validation of long-term safety and efficacy for smart materials. For clinical translation, multicenter trials are essential to verify the generalizability of novel diagnostic and therapeutic strategies. Multimodal data integration represents a critical future direction. Constructing knowledge graphs by synthesizing clinical, imaging, and biomolecular data will accelerate precision medicine research. Closed-loop systems integrating real-time monitoring, intelligent analytics, and precision interventions could enable comprehensive AL management spanning early warning → timely intervention → therapeutic efficacy evaluation.

## 8. Conclusion

In summary, the management of gastrointestinal AL is anchored in established minimally invasive endoscopic therapies, supported by an evolving evidence base. For the practicing clinician, these modalities form the current cornerstone of interventional management. The reviewed experimental technologies—from REGB and smart hydrogels to AI and microneedle platforms—represent a promising but speculative frontier. Their transformative potential is evident in preclinical models, but definitive proof of efficacy, safety, and cost-effectiveness in humans is awaited. The future path lies in the rigorous clinical validation of these innovations, and their thoughtful integration with proven strategies, to truly shift the paradigm towards proactive prevention and precision care.

## Data Availability

No new data were created or analyzed in this study. Data sharing is not applicable to this article.
